# Implementation of HER2 Testing in Endometrial Cancer, a Summary of Real-World Initial Experience in a Large Tertiary Cancer Center

**DOI:** 10.3390/cancers16112100

**Published:** 2024-05-31

**Authors:** Anna Plotkin, Ekaterina Olkhov-Mitsel, Weei-Yuarn Huang, Sharon Nofech-Mozes

**Affiliations:** 1 Department of Anatomic Pathology, Precision Diagnostics & Therapeutics Program, Sunnybrook Health Sciences Centre, Toronto, ON M4N 3M5, Canada; 2 Laboratory Medicine and Pathobiology, University of Toronto, Toronto, ON M5S 1A8, Canada

**Keywords:** endometrial cancer, HER2 amplification, HER2 expression, biomarker testing, *ERBB2* amplification, targeted therapy

## Abstract

**Simple Summary:**

In this study, scientists investigated how pathologists test for a protein called HER2 in a type of cancer called endometrial cancer (EC). They checked samples from 180 patients tested over the first two years following approval of the test in the clinic. They found that 28% of the samples had HER2 overexpression, which makes them eligible for HER2-targeted therapies. They also noticed that in some cases, the test results were different depending on the method used. This study shows that there are challenges in testing for HER2 in endometrial cancer and suggests that we need standardized guidelines to help doctors make better decisions about endometrial cancer treatment.

**Abstract:**

HER2-targeted therapies have transformed the management of advanced or recurrent serous endometrial cancer (EC), leading to an increased clinical demand for HER2 testing. Despite its adoption in select academic centers, the global extent of such tumor testing is unclear. In this study, we report on the initial two-year experience of HER2 testing at a major academic center with a reference gynecologic oncology service and biomarker reference laboratory. All patients who underwent HER2 testing based on physician discretion, reflex HER2 testing, and reference laboratory requests were included. From February 2021 to October 2023, HER2 testing was performed on 192 tumor tissue samples from 180 EC patients. Serous carcinoma constituted 52% of samples, reflecting diagnostic challenges and limited therapeutic options for advanced EC. HER2 positivity was found in 28% of all cases and 30% of p53-aberrant cases. An immunohistochemistry (IHC) score of 3+ was found in 15% of samples, while IHC 2+ was found in 45% (13% IHC 2+/ISH+ and 32% IHC 2+/ISH−). The newly identified ‘HER2-low’ category comprised 46% of the samples. Heterogeneity was noted in 42% of HER2-positive cases, with complex patterns in 3%. NGS and HER2 IHC-FISH showed a 24% discordance, attributed to intratumoral heterogeneity, tumor cellularity, a small number of amplified cells, and the HER2/CEP17 ratio near the cut-off. This study offers real-world insights into HER2 testing in EC, highlighting the challenges and underscoring the need for standardized guidelines in specimen handling, proficiency testing, and scoring criteria to enhance patient management and therapeutic decision-making.

## 1. Introduction

Endometrial cancer (EC) is the most common gynecologic malignancy in developed countries, with a global increase in incidence and mortality [1,2]. The endometrioid subtype of EC constitutes approximately 85% of cases, with surgical resection serving as the primary treatment modality, yielding a favorable prognosis, particularly in low-grade tumors and those detected at an early stage [3,4]. High-risk EC often requires adjuvant radiation and systemic therapy [4,5]. Treatment options for advanced and recurrent ECs are limited, with radiotherapy and chemotherapy showing limited efficacy [6]. In this regard, EC molecular classification introduced by The Cancer Genome Atlas has enhanced diagnostic accuracy and refined risk stratification, identifying patients who can benefit from targeted therapies, ushering in a paradigm shift in patient treatment options [7,8].

Human epidermal growth factor receptor 2 (HER2) overexpression, known to occur across a broad spectrum of solid tumors, enables targeted therapy with monoclonal anti-HER2 antibodies, such as trastuzumab, significantly improving patient survival [9]. Trastuzumab has become the mainstay in the treatment of patients with HER2-positive breast and gastroesophageal cancers [10]. In EC, HER2 overexpression varies from 4% to 69% in different studies, with a predominant occurrence in high-grade serous, carcinosarcoma, and clear cell histologies [11]. HER2 overexpression is correlated with the high copy number/p53-abnormal EC molecular subgroup and associated with a worse prognosis, even in early-stage disease [12,13]. A recent study further supports this association, indicating that HER2 overexpression is associated with an unfavorable prognosis [14].

In a landmark randomized phase 2 clinical trial in 2018 (NCT01367002), it was demonstrated that trastuzumab, in combination with carboplatin/paclitaxel, significantly improves progression-free and overall survival for HER2-positive advanced-stage and recurrent endometrial serous carcinomas (ESCs) compared to chemotherapy alone [15,16]. This was significant, given that ESC is an aggressive histological subtype of EC, associated with poor clinical outcomes, a high risk of recurrence, and a high mortality rate [17,18]. Subsequently, the National Comprehensive Cancer Network (NCCN) guidelines recommended the addition of trastuzumab to standard chemotherapy for advanced-stage or recurrent HER2-positive ESC [19,20], leading to an increasing clinical demand for HER2 testing in pathology laboratories across numerous jurisdictions, including Canada. While HER2 testing of EC is conducted as a standard of care in some academic centers, the global extent to which such tumor testing is performed remains unclear.

There is extensive experience in HER2 testing for breast and gastric carcinomas, in contrast to a notable lack of a standardized method for HER2 evaluation in EC, a critical step for precise patient triage and optimizing the selection of patients who may benefit from HER2-targeted therapy with monoclonal antibodies and, possibly, with antibody drug conjugates. This poses considerable challenges for pathologists in current clinical practice. Here, we audited our practice to bridge the knowledge gap by presenting a summary of real-world initial experience from a major academic center with a reference gynecologic oncology service and biomarker reference laboratory.

## 2. Materials and Methods

### 2.1. Study Population and Data Acquisition

This study was approved by the Sunnybrook Health Sciences Centre Research Ethics Board (SUN-5582) and written informed consent was waived. A retrospective search of the laboratory information system (Sunquest CoPath) was performed to identify ECs that underwent HER2 testing from the first case in February 2021 to October 2023. Clinicopathological features including the age at diagnosis, date of primary diagnosis, histological subtype and tumor grade, estrogen receptor (ER) and progesterone receptor (PR) status, MMR protein (MLH1, MSH2, MSH6, and PMS2) immunohistochemistry (IHC) results, p53-IHC status, and *POLE*-mutation and *ERBB2*-mutation statuses, when available, were recorded from the pathology reports for all patients.

### 2.2. HER2 Assessment by Immunohistochemistry and Fluorescence In Situ Hybridization

#### 2.2.1. General Considerations

Following the NCCN guideline recommendations for the addition of trastuzumab in stage III-IV or recurrent ESC treatment, initial HER2 testing requests at our institution were made by gynecologic and medical oncologists. Subsequently, Health Canada’s approval of trastuzumab for this indication and securing of funding within the Canadian public healthcare system prompted the implementation of reflex HER2 testing for in-house cases and cancer reviews and as a reference laboratory for external institutions.

#### 2.2.2. Tissue Samples

HER2 testing was initiated on the first available tissue in our laboratory with a preference to test formalin-fixed, paraffin-embedded (FFPE) endometrial biopsy material, assuming superior preanalytical conditions, minimal cold ischemic time, enhanced formalin penetration through fragmented tissue, and a shorter fixation time. In advanced cases with extrauterine disease, HER2 testing of such tissue was carried out. Cases with recurrent disease were tested based on the availability of tissue, namely, from the endometrial primary or metastatic site. Notably, 12 cases in our cohort had more than one site tested, including primary, recurrent, and/or metastatic sites.

#### 2.2.3. Histologic Subtyping

An upfront pathology review and assignment of the histological subtype were carried out by experienced gynecologic pathologists prior to HER2 testing. Carcinosarcoma, high-grade, and rare advanced/recurrent low-grade endometrioid cases, and occasionally cases with normal/wild-type p53 statuses, were tested for HER2 upon requests from clinicians. Cases with an uncertain primary site of origin were excluded from this audit.

#### 2.2.4. HER2 Testing by Immunohistochemistry

HER2 IHC staining was performed on whole-slide tissue sections using the Ventana 4B5 antibody (Roche) as per current standards validated for clinical practice (Supplementary Materials). HER2 IHC-stained slides were reviewed by one of two expert gynecologic and breast pathologists (A.P. or S.N-M.) and scored in accordance with the 2020 ISGyP recommendations [21].

Cases were scored as HER2 3+ if they exhibited strong basolateral/lateral or circumferential membranous HER2 staining in >30% of tumor cells. A score of 2+ (equivocal) was given if staining was seen in ≤30%, or weak to moderate membranous staining in ≥10%, of tumor cells, followed by reflex HER2 in situ hybridization (ISH) to confirm gene amplification. A score of 1+ was given if faint/barely perceptible, incomplete membrane staining was seen in any proportion, or weak complete staining in <10% of tumor cells. Cases were scored 0 when no staining was seen. For the purpose of this audit, cases were categorized as HER2-low if the tumor had an IHC score of 1+ or a non-amplified IHC score of 2+, but this category was not reported clinically. 

#### 2.2.5. HER2/Neu Testing by Fluorescence In Situ Hybridization

Cases with an equivocal IHC (score 2+) were tested by dual-probe fluorescence in situ hybridization (FISH) for HER2/neu amplification using one of two protocols that were validated in our clinical laboratory, depending on reagents’ availability, affected by the COVID-19 pandemic: (1) PathVysion HER-2 DNA Probe kit (Vysis, Dover, IL); (2) automated HER2 IQFISH pharmDx (Dako Omnis) assay, in accordance with the current standards validated for clinical practice (Supplementary Materials).

Whole sections were evaluated by experienced HER2 FISH reader gynecologic and breast pathologists (A.P. or S.N-M.) in accordance with the 2020 ISGyP recommendations [21]. HER2 amplification (IHC 2+/ISH+) was defined by a HER2/CEP17 ratio of ≥2.0 or average HER2 copy number of ≥6 per nucleus in a tight group of a minimum of 20 cells. Hematoxylin and eosin (H&E), IHC, and FISH slides were produced using sequential sections mounted in a similar orientation and evaluated together. The entire tissue section was visualized on a fluorescent microscope at low power using a DAPI filter to identify the tumor. The entire tumor was visualized using the orange filter (designed to detect HER2-gene signals) with special attention to the areas showing the highest IHC intensity. When variations in the number of HER2-gene signals occurred, pathologists performed formal enumeration in areas with the highest signal count number. The green filter (designed to detect CEP17 signals) was used for enumeration and computation of the FISH ratio.

#### 2.2.6. Heterogeneity

Cases were considered heterogenous if there was distinct region or regions (subclonal) of HER2 overexpression by IHC or FISH gene amplification [21,22]. The presence of scattered individually amplified nuclei that failed to form a tight cluster of at least 20 amplified cells was noted in the report, but these tumors were not considered amplified. In cases with heterogenous expression, the cases were considered amplified if at least one focus had at least 20 amplified cells.

### 2.3. ERBB2 Next-Generation Sequencing

*ERBB2* mutational status was recorded from molecular pathology reports. At our institution, targeted exome sequencing is performed as part of routine standard-of-care clinical testing on FFPE tumor tissue in all high-grade and/or p53-abnormal and/or MMR-deficient ECs with the S5XL next-generation sequencing (NGS) system and the Oncomine Comprehensive Assay, version 3 (OCAv3, Thermo Fisher Scientific Inc., Waltham, MA, USA), as per current standards validated for clinical practice. The OCAv3 panel detects *ERBB2* gene amplification, insertions/deletions, and single-nucleotide variants (SNVs). In brief, following evaluation of tumor adequacy, DNA was isolated from a macrodissected area, selected by the molecular pathologist based on H&E, independent of IHC and FISH. The *ERBB2* copy number ascertained by NGS was analyzed by the vendor’s bioinformatic pipelines (Ion Reporter Version 5.18, Thermo Fisher Scientific Inc., Waltham, MA, USA) and reported only if data QC thresholds met with the established standards. According to our in-house validation study, regardless of tumor cellularity, a cut-off of greater than 4 copies at a 5% CI (confidence interval) was considered positive for *ERBB2* amplification.

### 2.4. Statistical Analyses

Descriptive statistics were used to summarize the HER2 statuses and clinical characteristics of patients. The sensitivity, specificity, positive predictive value (PPV), and negative predictive value (NPV) were calculated for the *ERBB2* gene amplification status by NGS, comparing the results with the HER2 status by IHC-ISH. 

## 3. Results

### 3.1. Patient and Tumor Characteristics

From February 2021 to October 2023, a total of 192 tumor tissue samples from 180 patients were evaluated by HER2 IHC, and, in cases with an equivocal IHC score, FISH was performed to yield the combined (IHC-ISH) HER2 status. Detailed clinicopathological characteristics of the cohort are presented in Table 1. The median patient age at presentation was 67 years (range 32–92 years). Samples included 1 pleural effusion, 80 biopsies, and 111 resection specimens, comprising 167 primary ECs, 13 local recurrences, and 12 metastases. Serous carcinoma, either pure or as part of mixed endometrial carcinoma, comprised 52.1% (100/192) of the samples, with 47.9% being non-serous ECs. A high-grade, unspecified histologic type comprised 20.8% (40/192), carcinosarcoma 11.5% (22/192), endometrioid 10.4% (20/192), and undifferentiated endometrial carcinoma 1.6% (3/192) of the samples. Of note, one case of undifferentiated carcinoma arose against a background of serous carcinoma. Additionally, there were two cases of high-grade EC with aberrant p53 expression (1.0%) and one case each of clear cell carcinoma, endometrial gastric-type carcinoma, and endometrial mesonephric-like carcinoma (each at 0.5%, 1/192).

Among 12 cases with multiple blocks examined: 5 involved primary EC surgical specimens and subsequent biopsies of recurrent or metastatic disease; 4 included testing of both primary EC and synchronous regional metastasis; 1 had a diagnostic pre-surgical biopsy and local recurrence biopsy tested; 1 had a pre-surgical biopsy, primary resection, and metastatic tumor tested; and 1 involved a primary EC specimen and subsequent metastatic pleural effusion tested for HER2 (Table 2).

### 3.2. Evaluation of HER2/Neu Oncoprotein Overexpression and Gene Amplification by FISH

**Figure 1:**
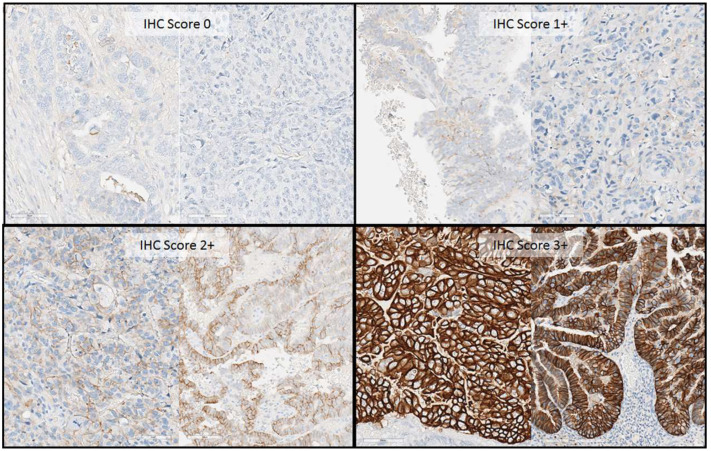
Immunohistochemical scoring of HER2. All photos derived from whole slide scanned image (40× resolution) displayed at 20× digital zoom. Score 0: No membranous labeling. Score 1+: Weak, barely perceptible membranous staining. Score 2+: Complete or incomplete membranous (including basolateral) staining with moderate intensity. Score 3+: Strong complete membranous (left) and strong basolateral staining.

A HER2 IHC score of 3+ was observed in 15.1% (29/192) of samples, while the equivocal HER2 expression (IHC score 2+) was identified in 44.8% (86/192) of samples (Table 3, Figure 1). Among these, IHC 2+ with a positive FISH assay (ISH+), defined as a HER2/CEP17 ratio of ≥2.0 or average HER2 copy number of ≥6 per nucleus in a tight group of a minimum 20 cells, constituted 13.0% (25/192), and IHC 2+ with a negative FISH assay (ISH−) accounted for 31.8% (61/192), of all samples (Table 3). The overall HER2 positivity rate, encompassing HER2 IHC-positive and/or HER2 FISH-amplified samples, was 28.1% (54/192). The remaining 71.9% of ECs (138/192) that did not overexpress HER2 were defined as HER2-negative based on a conventional binary classification. Notably, all cases were scored with a newly recognized category, ‘HER2-low’, defined as a HER2 IHC score of 1+ and/or IHC 2+/ISH−, accounting for 46.4% (89/192) of samples within our cohort. 

When assessing the 12 cases with multiple blocks examined, a discordant HER2 status was noted in 2 patients (16.7%). One case exhibited a positive HER2 status in the primary tumor and a negative status in a synchronous lymph node metastasis, while another case showed a negative HER2 status in the primary tumor and a positive status in the local recurrence in the fallopian tube, with heterogeneity observed in the latter (Table 2).

**Figure 2:**
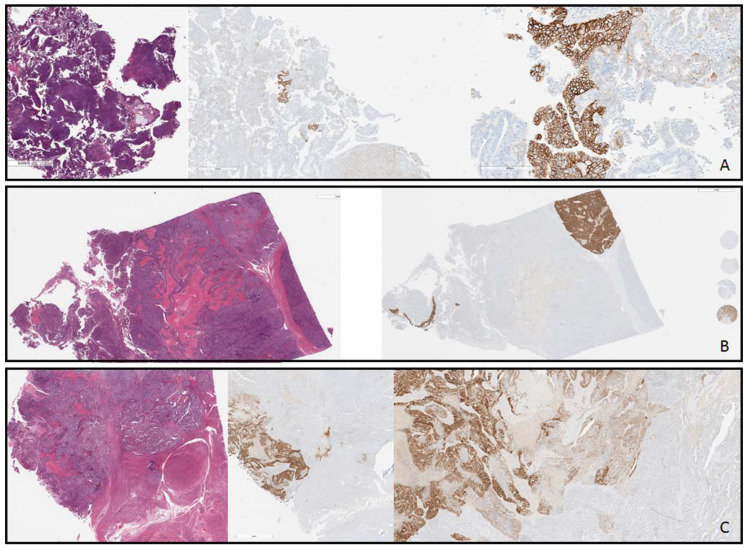
Examples of intratumoral heterogeneity. All three cases represent high-grade endometrial carcinoma, serous type, molP53abn. (**A**). Endometrial curettage specimen. There is strong complete membranous staining in less than 10% and weak to moderate basolateral staining in 10–20% corresponding to an immunohistochemical (IHC) score of 2+ (ISGyP criteria). Fluorescence in situ hybridization (FISH) analysis demonstrates HER2/neu gene amplification (FISH ratio is 2.2 confined to areas of strong complete membranous staining). *ERBB2* mutational analysis is negative for SNV or INDEL. (**B**). Hysterectomy specimen. There is strong complete membranous staining in just over 10% against a background of essentially no staining in the remaining tumor corresponding to an IHC score of 2+ (ISGyP criteria). FISH analysis demonstrates HER2/neu gene amplification (FISH ratio is 5.7 confined to areas of strong complete membranous staining). Note on slide external control representing the full range of IHC scores. (**C**). Hysterectomy specimen. There is strong complete membranous staining in 10–20% and weak to moderate basolateral staining in 10–20% corresponding to an IHC score of 2+ (ISGyP criteria). FISH analysis demonstrates HER2/neu gene amplification (FISH ratio is 4.2 confined to areas of strong complete membranous staining and 2.2 in areas with moderate intensity).

In 38.3% (44) of 115 samples with HER2-positive and equivocal scores (IHC 3+ and 2+), regional heterogeneity was noted. Additionally, 4 (3.5%) cases had complex heterogeneous staining patterns including scattered individual amplified cells that did not form a tight cluster of 20 cells [23]. Among 62 samples with a HER2/CEP17 ratio of <2.0, 3 had rare cells with HER2 copy numbers ≥6 per nucleus. Examples of intratumoral heterogeneity are illustrated in Figure 2A–C.

Seven samples were reviewed by two readers, all IHC 2+/ISH+. In cases with available p53 IHC data, the majority (87.9%, 152/173) displayed aberrant p53 staining patterns. None of ECs with a wild-type p53 immunophenotype were HER2-positive, 60.0% (12/20) were HER2-negative (8/20), and 40% were HER2-low (Table 3).

A small percentage, constituting 3.0% (5/165) of the cases tested, exhibited MMR deficiency, all of which were HER2-negative. Additionally, 1.2% (2/165) showed subclonal loss of MMR, with one being HER2-positive. Among the cases with multiple classifiers, three demonstrated concurrent MMR deficiency and p53 abnormality (with 2/3 cases exhibiting subclonal p53 loss). Additionally, four cases had *POLE* mutations, and three had variants of uncertain significance, all of which were HER2-negative (Table 3). All *POLE*-mutated cases were p53-abnormal by IHC, one was MMR-deficient, and one had subclonal loss of MMR.

### 3.3. Correlation between HER2 Testing by IHC-ISH and ERBB2 Determined by Next-Generation Sequencing

Among 92 endometrial carcinomas analyzed by NGS and HER2 IHC-FISH, 10 (10.9%) had *ERBB2* gene amplification by NGS, all of which were HER2-positive by IHC-FISH. Two cases with variants of unknown significance (VUS) were identified, both IHC 2+/ISH−. Furthermore, 80 cases demonstrated a lack of *ERBB2* gene amplification, SNVs, insertions, or deletions according to NGS analysis. Among these cases, 29 were classified as IHC 0, 11 as IHC 1+, 18 as IHC 2+/ISH−, 13 as IHC 2+/ISH+, and 9 as IHC 3+. Consequently, discordance between IHC-ISH and NGS was observed in 23.9% (22/92) of cases, which can be attributed to intratumoral heterogeneity (Figure 2A), tumor cellularity, a small number of amplified cells, and a HER2/CEP17 ratio close to the cut-off (Table 4). The sensitivity, specificity, positive predictive value, and negative predictive value of the *ERBB2* gene amplification status ascertained by NGS were 31.3%, 100%, 100%, and 73.2%, respectively, compared with HER2 IHC-ISH.

## 4. Discussion

The effectiveness of trastuzumab therapy in HER2-positive advanced or recurrent ESC has established HER2 as a pivotal predictive biomarker, emphasizing the need for detailed validated and standardized testing strategies. The current study is among the largest audits, to date, of the HER2 status in a real-world clinical setting of EC. In the absence of consensus recommendation guidelines for HER2 testing in EC addressing different IHC and ISH scenarios, our initial practical implementation of the testing algorithm prompted various questions to be raised, especially in cases with heterogeneous, nuanced, and/or uncommon staining patterns.

For the interpretation of IHC staining, three primary sets of guidelines are used in clinical practice, namely the ISGyP recommendations [21,24,25], the 2018 ASCO/CAP guideline recommendations for HER2 testing in breast carcinoma [26], and FDA guidance [27,28]. The IHC score is determined by evaluating subcellular localization (membranous), circumferential versus incomplete staining, intensity, and the percentage of tumor cells exhibiting this pattern. The criteria used in the clinical trial (NCT1367002), leading to trastuzumab’s approval and the formulation of associated NCCN guidelines, were based on the ISGyP modified 2013 ASCO/CAP HER2 testing recommendations in breast carcinoma, defining IHC 3+ as strong, complete circumferential or basolateral membranous staining in 30% of neoplastic cells [16]. In contrast, the 2018 ASCO/CAP breast cancer guidelines and FDA guidance define IHC 3+ as complete circumferential membranous staining that is intense in at least 10% of tumor cells. While most IHC results would be concordant regardless of the criteria used for interpretation, certain cases, with more than 10 but less than or equal to 30% circumferential labeling, would be considered 3+ by the 2018 ASCO/CAP but equivocal by ISGyP. Similarly, cases with intense basolateral labeling >30% may be considered 3+ by ISGyP but equivocal by the 2018 ASCO/CAP. Notably, the 2023 ASCO/CAP guidelines for HER2 testing in EC, largely in line with ISGyP, define IHC 3+ as intense complete or basolateral/lateral membrane staining in >30% of tumor cells; 2+ as intense complete or basolateral/lateral membrane staining in <30%, or weak to moderate staining in ≥10% of tumor cells; 1+ as incomplete membrane staining that is faint/barely perceptible in any proportion of cells or weak complete staining in <10% of tumor cells; and 0 as no staining [29]. Most discrepant cases are expected to be adjudicated by FISH, although occasional cases may result in a discordant IHC-ISH category. Hashem et al. [30] compared the two scoring methods and found comparable results for HER2-positive cases and 98% concordance of all HER2 IHC and/or FISH results. Differences in guidelines further include the HER2-low category, wherein the 2018 ASCO/CAP breast cancer guidelines include faint membranous labeling in ≤10% within a score of 0, whereas ISGyP and FDA guidance defines a score of 0 as absolutely no staining. Interest in the lower end of the HER2 expression spectrum has increased due to the effectiveness of HER2 antibody–drug conjugates (ADCs) in HER2-low breast cancer [31]. EC is also being studied in this context in ongoing clinical trials (NCT04482309, NCT04585958, NCT05150691). As new trial data emerge, refinements to HER2 IHC interpretation criteria and anti-HER2 treatment options are expected.

The ISGyP, 2018 ASCO/CAP, and FDA guidelines also differ in HER2 FISH interpretation. The NCT01367002 trial consider tumors with ≥20 contiguous nuclei and a HER2 FISH score ≥2 as amplified (according to a personal communication with Dr. Buza) [16]. This is in line with the original test package insert sheet filed with the FDA for the setting of breast carcinoma [32]. In contrast, the 2018 ASCO/CAP guidelines define amplification as ≥10% of the tumor having a positive FISH score. Furthermore, ISGyP and FDA guidance recognize only two FISH scoring categories based on a HER2/CEP17 signal ratio cut-off of 2, whereas the 2018 ASCO/CAP recognizes five FISH categories. Notably, in the latter, tumors with a HER2 gene copy number ≥6 and a HER2/CEP17 ratio ≥2 are categorized as amplified. In our cohort, two HER2 IHC 2+ cases had a HER2/CEP17 ratio <2 and one had rare single cells with a HER2 copy number ≥6, while another had scattered neoplastic cells with amplification (HER2 copy number 2–8, CEP17 copy number 2–4) but lacked a cluster with a FISH ratio ≥2 in 20 contiguous cells, resulting in a classification of not amplified for both cases. Another case exhibited strong basolateral and complete membranous HER2 IHC staining in 40% of the well-fixed surface area. The FISH (performed for QA purposes) HER2/CEP17 ratio was <2.0 and the average HER2 copy number was ≥6.0 signals/cell. Following the ISGyP guidelines, this would be considered non-amplified, while when using the 2023 ASCO/CAP guidelines, this would be considered positive. In view of the IHC analysis, the overall HER2 status of this tumor was summarized as positive. Further clinical investigations are required to evaluate the correlation between specific HER2 FISH result categories and its impact on therapeutic response in EC.

In the audit presented here, the HER2 IHC/FISH-derived status was positive in 28.1% and 30.2% of all cases and p53-aberrant cases, respectively, akin to the proportion reported in the NCT1367002 clinical trial, which demonstrated a survival benefit for anti-HER2 therapy in ESC [16]. In a comparative study of over 1500 cases focusing on testing platforms and interpretation criteria in uterine serous carcinoma, the HER2 positivity rate varied from 10.5% to 19.6%, with the lowest number detected by NGS and the highest by FISH using the 2018 ASCO/CAP breast cancer guidelines [33]. The authors did not specify whether they analyzed biopsies or resections, the extent of FISH analysis (whether it covered the entire tumor surface or a limited segment), or if tissue microarrays were used. Furthermore, it is unclear whether IHC and ISH were scored by the same pathologist or another molecular pathologist or scientist. In another study of 93 uterine serous carcinomas, of which 71 were tested on resected specimens using the 2018 ASCO/CAP criteria, only 4% had an IHC score of 3+, 25% had a FISH ratio ≥2, and 4% were deemed amplified based on a HER2 copy number ≥6 [34]. In this series, one case with IHC score of 1+ was amplified by FISH. These factors suggest that preanalytical parameters, most importantly the prolonged cold ischemic time (implicated in resected specimens due to tissue penetration and handling), regional heterogeneity, and the extent of amplified nuclei required for reporting FISH positivity may contribute to the observed differences. 

Significant intratumoral heterogeneity in HER2 protein expression and gene amplification has been noted in ESC [35]. In our cohort, 38.3% (44/115) samples with HER2-positive and equivocal scores (IHC 3+ and 2+) exhibited regional heterogeneity and 3.5% demonstrated complex patterns, including scattered individual amplified cells and spatially distinct amplification, as previously described [35]. This was most prevalent with HER2 IHC 2+ scores. Another study found that over 50% of HER2-positive ESCs exhibited significant intratumoral heterogeneity, emphasizing the need for standardized definitions [20]. Previous research has identified the challenges of HER2 heterogeneity in breast tumors and its implications for treatment eligibility [36]. Building on this, larger studies are also needed for a comprehensive understanding of the clinical impact of intratumoral heterogeneity in upcoming clinical trials of anti-HER2-targeted therapies in EC. Furthermore, the heterogeneous nature of HER2 staining in ESC prompts considerations about the optimal specimen/s type for testing, including biopsy versus hysterectomy, primary versus recurrent and metastatic lesions, and whole-tumor sections versus specific regions, as limited data currently exist. In our audit, a discordant HER2 status between primary and corresponding extrauterine lesions was observed in 16.7% of cases. Technical issues, like suboptimal reproducibility of HER2 staining and scoring, may have contributed to the observed discrepancies, while tumor heterogeneity could also have played a role. This emphasizes the potential benefit of sampling biopsies from multiple primary, recurrent, and metastatic sites in certain cases to optimize individualized treatment. Furthermore, we advocate for the same subspecialty pathologist, with expertise in the field, to interpret both IHC and FISH, scoring the entire slide, acknowledging that in many laboratories, ISH is often assessed by molecular pathologists, cytogeneticists, or scientists based on selected (encircled) tumor areas. 

The unresolved practical considerations in the HER2 testing algorithm for EC warrant further investigation. Pathology laboratories should adopt evidence-based standardized universal protocols for EC specimen handling and fixation, aligning with established guidelines for breast and gastric carcinomas [20]. Dual-probe HER2 FISH signal degradation over time and under different slide storage conditions should be considered. Storing slides at −80 °C has been shown to allow for signal retention, while at room temperature, signals start to degrade, with CEP17 signal loss at a faster rate [37]. Additionally, laboratories should participate in quality assurance and proficiency testing programs specifically for HER2 IHC and ISH in EC, which need to be established. Scoring HER2 would benefit from designated readers with appropriate training and experience. Preferably, professional bodies should recommend minimum annual performance and interpretation standards for competency (to allow familiarity with the many nuances of test interpretation). Pathologists should carefully consider the added implications of EC histological subtyping and the critical importance of selecting appropriate tissue blocks for HER2 testing in the context of targeted therapy, especially in cases with mixed and ambiguous histologies. 

The NCT1367002 clinical trial, which demonstrated a survival benefit for anti-HER2 therapy, focused on the serous subtype, because of its aggressiveness, unfavorable prognosis, and notably higher rate of HER2 overexpression [15,16,38]. Our audit examined an unselected, real-world cohort of EC patients undergoing HER2 testing at a major academic center with a reference gynecologic oncology service and biomarker reference laboratory. The findings align with prior studies, confirming HER2 overexpression in various high-grade EC histotypes, supporting the potential extension of this therapeutic strategy to other EC subtypes in the future [39,40]. Currently, four ongoing trials, including NRG-GY026 (NCT05256225), are investigating HER2-targeted therapies in both serous and non-serous EC, covering advanced stages, recurrence, metastasis, and p53-abnormal high-grade EC. Additionally, five trials focus on HER2-targeted therapies in HER2-positive solid tumors, with a subgroup emphasis on EC. Recently, the FDA has granted accelerated approval to fam-trastuzumab deruxtecan-nxki (Enhertu, Daiichi Sankyo, Inc., Tokyo, Japan) for adult patients with unresectable or metastatic HER2-positive (IHC 3+) solid tumors who have received prior systemic treatment and have no satisfactory alternative treatment options, underscoring the need for clear testing guidance [41].

At our institution, EC tissues that undergo molecular profiling by NGS are assessed with the OCAv3 assay for *POLE* tumor profiling for EC molecular classification. The OCAv3 assay includes the *ERBB2* gene, allowing for the detection of gene amplification and mutations by NGS [42]. Despite the current reliance on IHC-FISH for HER2 testing, there is increasing interest in utilizing NGS for a more cost-effective and tissue-efficient evaluation of *POLE* and *ERBB2* alterations in EC, especially in cases with limited material. Studies have linked *ERBB2* amplifications detected by NGS to the effects of trastuzumab, pertuzumab, and lapatinib in breast cancer [43]. To date, clinical validation of *ERBB2* assessment by NGS methods in EC is lacking, and its concordance with traditional methods, as well as its association with the effects of targeted anti-HER2 therapy, remains to be established. Previous studies have shown strong concordance between NGS and IHC-FISH in breast and gastric tumors [44]. Robinson et al. [34] reported 100% concordance between NGS and combined IHC-FISH for identifying *ERBB2* amplification in ESC, though the small fraction of HER2-positive cases and the limited study sample size may limit the generalizability of their findings. In our cohort, discordance between IHC-FISH and NGS was observed in 23.9% of cases, possibly attributable to factors such as the low tumor content, small size of tumors with HER2 overexpression/amplification, heterogeneity, and low-level amplification, resulting in false-negative results or low-level copy number gains. Specifically, intratumoral heterogeneity may have contributed to this discordance, as NGS provides an aggregate measurement over the tumor area and may not detect amplification present only within a cell subpopulation. Of note, we did not evaluate different thresholds for calling *ERBB2* amplification by OCAv3 to establish optimal concordance with FISH. Our NGS cut-off for *ERBB2* amplification is set at >4 copies (Minimum Ploidy Gain, 5% CI >4), and taking tumor contents and intratumoral heterogeneity into consideration, lowering the cut-off to ≥3 copies could potentially improve the concordance rate from 76.1% to 88.0%. Therefore, further optimization and standardization of criteria for NGS-based detection of *ERBB2* amplification in EC are needed to potentially improve the concordance with FISH. Nevertheless, from a clinical standpoint, IHC remains the preferred primary HER2 testing modality, as it enables a visual assessment of intratumor heterogeneity and, if needed, can be performed on multiple tissue blocks at a relatively low cost. 

The present audit of our initial experience with HER2 testing in EC has several limitations. The retrospective design of the audit restricted our ability to assess the clinical response to anti-HER2 therapy or investigate predictive and prognostic biomarkers. We do not have information on the proportion of HER2-positive EC patients that were considered for trastuzumab therapy or outcome data for this patient population. Additionally, FISH was exclusively conducted on HER2 IHC 2+ cases, limiting a comprehensive assessment of the concordance between HER2 IHC, FISH, and NGS in EC. Nevertheless, our findings provide insights into the practical challenges of implementing HER2 testing for EC in the clinical setting. We encountered questions regarding specimen selection, repeat testing in recurrent/metastatic tumors, and the effective management of associated spatiotemporal heterogeneity.

## 5. Conclusions

In conclusion, our implementation of HER2 testing for EC in a major academic center emphasizes lingering practical questions about the testing algorithm. It underscores the need for standardized universal guidelines and requirements concerning specimen handling, proficiency testing, and the use of scoring criteria. Professional expertise is essential for scorers in this context. In an unselected population, nearly half of the requests for HER2 testing occurred in non-serous EC cases, reflecting the notion that histologic subtyping of high-grade EC is challenging as well as the limited management options for advanced EC. The HER2 intratumoral heterogeneity observed in our cohort, along with in previous studies, underscores the need for further research into its role in trastuzumab resistance. IHC-ISH proved more sensitive than NGS in identifying HER2-positive EC cases in our institution, with factors such as intratumoral heterogeneity influencing the reduced HER2 amplification detection by NGS. Future studies are needed to establish the optimal concordance with IHC-FISH and evidence-based guidelines for HER2 testing.

## Figures and Tables

**Table 1:** Clinical and pathological characteristics of endometrial cancer patients tested for HER2 status, 2021–2023.

Characteristic	*N* (%)
**Age** (range, years)	67 (32–92)
**Specimen Type**	
Biopsy	80 (41.7)
Resection	111 (57.8)
Pleural effusion	1 (0.5)
**Tumor type**	
Primary	167 (87.0)
Local recurrence	13 (6.8)
Metastasis	12 (6.3)
**Histologic type**	
Serous	95 (49.5)
Mixed (with serous component)	5 (2.6)
Mixed (without serous component)	2 (1.0)
Carcinosarcoma	22 (11.5)
High-grade, unspecified, or ambiguous	40 (20.8)
Endometrioid	20 (10.4)
Endometrial adenocarcinoma with aberrant p53	2 (1.0)
Clear cell	1 (0.5)
Endometrial gastric-type carcinoma	1 (0.5)
Endometrial mesonephric-like carcinoma	1 (0.5)
Undifferentiated	2 (1.0)
Undifferentiated (with serous component)	1 (0.5)
**ER IHC**	
Positive	78 (52.0)
Low positivity *	32 (31.3)
Negative	40 (26.7)
N/A ^†^	
**MMR IHC**	
Intact	158 (95.8)
Deficient	5 (3.0)
Subclonal loss	2 (1.2)
N/A	27
**p53 IHC**	
Aberrant	152 (87.9)
Wild type	20 (11.6)
Equivocal	1 (0.6)
N/A	19
***POLE* status**	
Mutated	4 (3.7)
VUS	3 (2.8)
Wild type	101 (93.5)
N/A	84
**HER2 IHC/ISH score**	
0	49 (25.5)
1+	28 (14.6)
IHC 2+/ISH−	61 (31.8)
IHC 2+/ISH+	25 (13.0)
3+	29 (15.1)
**Heterogeneity**	
Yes	43 (22.4)
No	64 (33.3)
Special cases ^#^	3 (1.6)

* Low positivity was defined as <10%, including focal and very focal staining. ^#^ Special patterns included the following: (1) A case with weak to moderate complete membranous and basolateral HER2 staining in <5%. Rare scattered amplified cells (FISH ratio > 2), though these cells did not form an amplified cluster of 20 cells and overall did not exceed 1% when the entire tumor was scanned. (2) An unusual case with a borderline HER2 status. IHC showed 10–15% strong membranous staining that would be sufficient for classification of this tumor as IHC-positive based on criteria used for breast carcinoma. Yet, based on ISGYP recommendations, the IHC score using endometrial carcinoma criteria was 2+. Further complicating the issue was the absence of HER2/neu gene amplification by FISH. (3) A case with scattered individual (low) amplified cells noted, but these did not form an amplified region. ^†^ N/A not available.

**Table 2:** Audit of HER2 expression patterns in cases with multiple examined blocks.

Case	Specimen	Tumor	Histology	HER2 IHC/ISH	Hetero-geneity	Specimen	Tumor	HER2 IHC/ISH	Hetero-geneity	Specimen	Tumor	HER2 IHC/ISH
1	Biopsy	Primary	Serous	2+/ISH−	No	Resection	LN metastasis	2+/ISH−	No			
2	Biopsy	Primary	Serous	2+/ISH−	Yes	Biopsy	LR (Fallopian tube)	2+/ISH+	Yes			
3	Biopsy	Primary	Endometrioid	2+/ISH−	No	Resection	Primary	2+/ISH−	Yes	Biopsy	Metastasis (Lung)	0
4	Resection	Primary	Serous	2+/ISH+	Yes	Biopsy	Metastasis (Pancreas)	2+/ISH+	No			
5	Biopsy	Primary	Serous	0		Resection	LN metastasis	1+				
6	Resection	Primary	Endometrioid	1+		Pleural effusion	Metastasis	0				
7	Resection	Primary	HG, unspecified	2+/ISH−	No	Biopsy	LR (Omentum)	2+/ISH−	No			
8	Resection	Primary	HG, unspecified	0		Biopsy	LR (Peritubal tissue)	0				
9	Resection	Primary	Serous	2+/ISH−	Yes	Resection	LN metastasis	2+/ISH−	Yes			
10	Resection	Primary	Carcinosarcoma	1+		Biopsy	Metastasis (Kidney)	2+/ISH−	No			
11	Resection	Primary	Carcinosarcoma	2+/ISH−	Yes	Biopsy	LR (Peritoneum)	1+				
12	Resection	Primary	HG, ambiguous	3+		Resection	LN metastasis	0				

HG, high-grade; LN, lymph node; LR, local recurrence.

**Table 3:** Stratification of HER2 status based on histologic type, p53, MMR, ER, and pathogenic *POLE* mutation status.

	HER2				
	0	1+	IHC 2+/ISH−	IHC 2+/ISH+	3+
**Histologic Type**					
Serous	19 (20.0)	13 (13.7)	28 (29.5)	16 (16.8)	19 (20.0)
Mixed (with serous component)	2 (40.0)	-	2 (40.0)	1 (20.0)	-
Mixed (without serous component)	-	-	1 (50.0)	1 (50.0)	-
Carcinosarcoma	4 (18.2)	3 (13.6)	8 (36.4)	4 (18.2)	3 (13.6)
High-grade, unspecified	10 (33.3)	5 (16.7)	11 (36.7)	1 (3.3)	3 (10.0)
High-grade, ambiguous	1 (10.0)	-	3 (30.0)	2 (20.0)	4 (40.0)
Endometrioid	10 (50.0)	6 (30.0)	4 (20.0)	-	-
Endometrial adenocarcinoma with aberrant p53	-	1 (50.0)	1 (50.0)	-	-
Clear cell	-	-	1 (100)	-	-
Endometrial gastric-type carcinoma	-	-	1 (100)	-	-
Endometrial mesonephric-like carcinoma	-	-	1 (100)	-	-
Undifferentiated	2 (100)	-	-	-	-
Undifferentiated (with serous component)	1 (100)	-	-	-	-
**ER IHC**					
Positive	17 (21.3)	14 (17.5)	21 (26.3)	11 (13.8)	17 (21.3)
Low positivity	8 (27.6)	4 (13.8)	10 (34.5)	4 (13.8)	3 (10.3)
Negative	13 (32.5)	3 (7.5)	17 (42.5)	4 (10.0)	3 (7.5)
N/A ^†^	10 (23.8)	7 (16.7)	13 (31.0)	6 (14.3)	6 (14.3)
**MMR IHC**					
Intact	40 (25.3)	23 (14.6)	49 (31.0)	23 (14.6)	23 (14.6)
Deficient	2 (40.0)	1 (20.0)	2 (40.0)	0	0
Subclonal loss	1 (50.0)	0	0	1 (50.0)	0
N/A	6 (22.2)	4 (14.8)	10 (37.0)	1 (3.7)	6 (22.2)
**p53 IHC**					
Aberrant	35 (23.0)	21 (13.8)	50 (32.9)	23 (15.1)	23 (15.1)
Wild type	12 (60.0)	4 (20.0)	4 (20.0)	0	0
Equivocal	0	0	0	0	1 (100)
N/A	2 (10.5)	3 (15.8)	7 (36.8)	2 (10.5)	5 (26.3)
***POLE* status**					
Mutated	2 (50.0)	1 (25.0)	1 (25.0)	0	0
VUS	2 (66.7)	0	1 (33.3)	0	0
Wild type	29 (28.4)	13 (12.7)	24 (23.5)	15 (14.7)	21 (20.6)
N/A	16 (19.3)	14 (16.9)	35 (42.2)	10 (12.0)	8 (9.6)
**Total**	49 (25.5)	28 (14.5)	61 (31.8)	25 (13.0)	29 (15.1)

^†^ N/A not available.

**Table 4:** Details of immunohistochemistry (IHC), fluorescence in situ hybridization (FISH), and next-generation sequencing (NGS) in discordant HER2 cases.

Specimen	Tumor	Histology	IHC Score	FISH HER2/CH17 Ratio	Heterogeneity	HER2 NGS	Tumor Cellularity
Biopsy	Primary	Mixed (Serous, CC)	2+	9/3; 3	Y	3.79	40
Biopsy	LR	HG, unspecified	2+	6.5/2.2; 2.954	Y	3.08	40
Resection	Primary	HG, ambiguous	3+	6.1/5.5; 1.1	Y	1.94	75
Biopsy	Primary	Serous	2+	6/2; 3	Y	3.99	50
Resection	Primary	HG, ambiguous	2+	8/2.3; 3.478	Y	2.29	40
Resection	LR	Serous	2+	N/A ^†^	Y	2.66	60
Biopsy	Primary	Mixed (HG EC, LCNECa)	2+	4.5/2.2; 2.045	Y	1.93	75
Biopsy	Primary	Serous	2+	5.2/2; 2.6	Y	1.50	85
Biopsy	Primary	HG, ambiguous	2+	5.5/2.5; 2.2	Y	2.64	70
Biopsy	Primary	Carcinosarcoma	2+	4.6/2; 2.3	Y		Conducted off site
Biopsy	Primary	Serous	2+	6/2.5; 2.4	Y	2.58	70
Resection	Primary	Serous	2+	7.4/2.1; 3.5238	Y	2.21	80
Biopsy	Primary	Serous	2+	4.8/2.2; 2.2	Y	2.63	70
Biopsy	Primary	Carcinosarcoma	2+	5.2/2; 2.6	Y	3.64	25
Resection	Primary	HG, ambiguous	3+	-	Y	4.23	75
Resection	Primary	Serous	3+	3.5/2; 1.75	Y, special pattern	3.49	75
Resection	Primary	HG, ambiguous	3+	-	Y	3.72	45
Resection	Primary	Serous	3+	-	Y	4.40	80
Resection	Primary	HG, unspecified	3+	-	Y	2.17	20
Biopsy	Primary	Serous	3+	-	N		Conducted off site
Resection	M	Serous	3+	-	N	3.69	80
Biopsy	Primary	Serous	3+	-	N	3.02	10

^†^ N/A not available.

## Data Availability

The data that support the findings of this study are available on request from the corresponding author, A.P.

## Supplementary Materials

The following supporting information can be downloaded at https://www.mdpi.com/article/10.3390/cancers16112100/s1. Protocol for HER2 IHC, Protocol for HER2 ISH.
