# Supplementary material for: Implementation of HER2 Testing in Endometrial Cancer, a Summary of Real-World Initial Experience in a Large Tertiary Cancer Center
Source: Cancers (Basel). 2024 May 31;16(11):2100. doi: 10.3390/cancers16112100 (PMC11171265; doi:10.3390/cancers16112100)
Supplement: Supplementary file 1 [file cancers-16-02100-s001.zip › cancers-3014638-supplementary.pdf]

Protocol for HER2 IHC

**Immunostainer**	
Type:	Ventana Benchmark Ultra
**Primary antibody**	
Clone:	PATHWAY (clone 4B5)
Producer:	Ventana
Product no. / lot no.:	790-2991 / K14565
Format:	Ready-To-Use (prediluted)
Incubation time / temperature:	16 min. / 36°C
**Epitope retrieval, HIER**	
Device:	On Board / On Machine
Buffer:	Ventana CC1
Heating time at max. temp.:	36 min.
Maximum heating temp.:	95°C
**Visualization system**	
Producer:	Ventana
Product / no:	UltraView Universal DAB Detection Kit / 760-500
Incubation time polymer:	8 min.
Incubation temperature:	8°C
**Chromogen**	
Producer:	Ventana
Product / no:	ultraView Universal DAB Detection Kit / 760-500
Incubation time / temperature:	8 min. / 36°C
Enhancement:	CuSO4

Protocol for HER2 ISH

Target and stainer platform

Target:	HER2/CHR17 FISH
Stainer platform:	Dako Omnis

Assay

Assay:	SureFISH
Assay producer:	Dako/Agilent
Assay product no:	G110104R-8/G110105G-

Heat Induced Epitope Retrieval (HIER)

HIER device:	On Board / On Machine
HIER buffer:	ISH Pre-Treatment Solution
HIER buffer producer:	Dako/Agilent
HIER buffer product no:	GM30111-2
HIER time:	30 min.
HIER temperature:	97°C

Proteolysis

Enzyme:	ISH Pepsin
Enyzme producer:	Dako/Agilent
Enzyme product no:	GM30211-2
Proteolysis time:	30 min.
Preteolysis temperature:	32°C

Denaturation and hybridization of HER2 probe

Denaturation time:	10 min.
Denaturation temperature:	66°C
Hybridization time:	120 min.
Hybridization temperature:	45°C
